# Supplementary material for: Women’s Well-Being and Rural Development in Depopulated Spain
Source: Int J Environ Res Public Health. 2020 Mar 17;17(6):1966. doi: 10.3390/ijerph17061966 (PMC7143739; doi:10.3390/ijerph17061966)
Supplement: Supplementary File 1 [file ijerph-17-01966-s001.pdf]

Prof. Dr. Mª Teresa Terrón Caro, Professor of Facultad de Ciencias Sociales, Universidad Pablo de Olavide, director of rupo de Investigación en Acción Socioeducativa, GIAS (code HUM929) and specialist in the subject, states that:

After an external review of the instrument for collecting information prepared by Professor Vicente Llorent-Bedmar and Professor Verónica Cobano-Delgado Palma of the Universidad de Sevilla, and taking into account that the object of study of the research is people, so there is no need for ethical criteria in experiments or in the field of health sciences or with animals. At the same time, the people are not minors, so as they are adults, no elements are required to be followed in the case of research involving minors.

Bearing in mind the above and in accordance with the principles and ethical notions established by European, national and international regulations, I would like to inform you that:

The questionnaire "Youth and gender in rural areas. A socio-educational perspective", intended for Rural Development Groups (RDG), meets the requirements for ethical approval of research with human beings.

Review that was carried out on June 3, 2019.

TERRON CARO MARIA TERESA - 48812658B

Firmado digitalmente por TERRON CARO MARIA TERESA - 48812658B
Fecha: 2020.03.16 17:22:32 +01'00'

Fdo. María Teresa Terrón Caro

Director of Grupo de Investigación en Acción Socioeducativa

Universidad Pablo de Olavide Departamento de

Educación y Psicología Social Carretera de Utrera, Km.

1. C.P. 41013, Sevilla (España)
